# Supplementary material for: Impact of Ultrasound on a Gluten-Free Composite Flour Based on Rice Flour and Corn Starch for Breadmaking Applications
Source: Foods. 2025 Mar 21;14(7):1094. doi: 10.3390/foods14071094 (PMC11988527; doi:10.3390/foods14071094)
Supplement: Supplementary file 1 [file foods-14-01094-s001.zip › foods-3514093-supplementary.pdf]

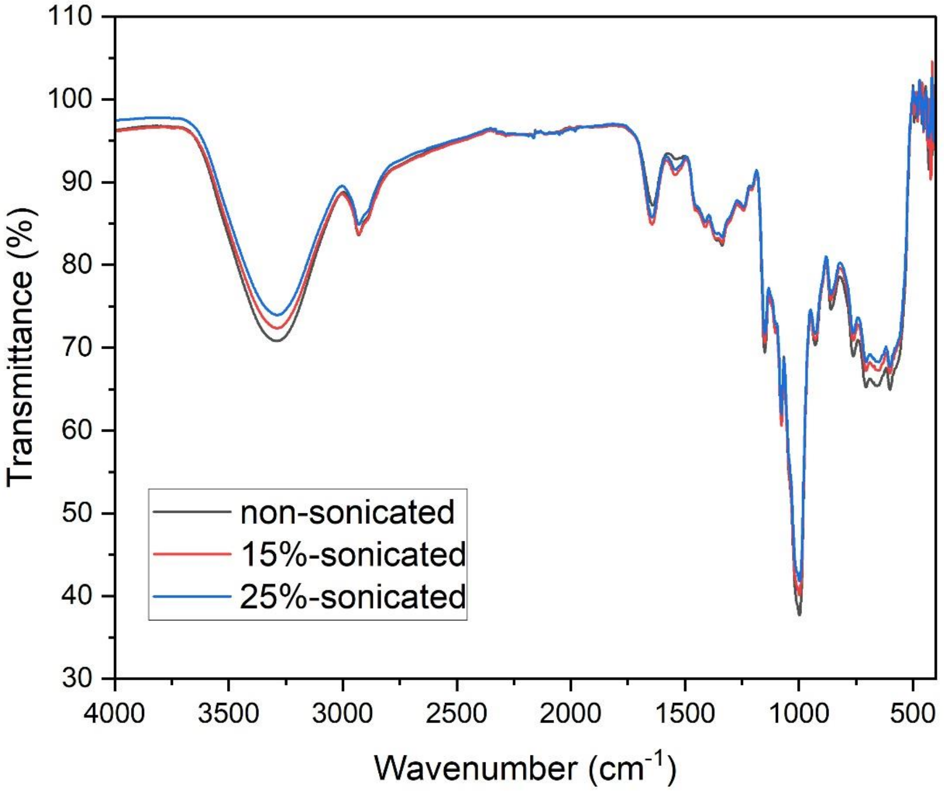

Figure S1. FTIR Spectra of the non-sonicated (black line), 15%-sonicated (red line), and 25%-sonicated (blue line).
